# Investigating the dietary intentions of Iranian tourists regarding the consumption of local food

**DOI:** 10.3389/fnut.2023.1226607

**Published:** 2023-11-16

**Authors:** Maryam Mohammadian Pouri, Mehdi Rahimian, Saeed Gholamrezai

**Affiliations:** Department of Agricultural Economics and Rural Development, Lorestan University, Khorramabad, Iran

**Keywords:** tourism, local food, intention, risk perception, TPB

## Abstract

**Introduction:**

Attention to local food among tourists is increasing day by day. However, studies on the behavior and intention of tourists towards consuming these foods are few. Limited studies in this field prompted us to help fill the research gap by doing this research.

**Methods:**

This study aims to use the extended theory of planned behavior (TPB) by adding risk perception (RP) to investigate the tourists’ intentions towards local food (TILF) in Iran. The extended model tested 313 tourists visiting local food supplies.

**Results:**

Results show that the extended TPB explains 87.8% of tourist intention variance. Three variables of the original TPB had positive impacts on tourists’ intentions. A notable result of this study was the realization of a negative relationship between RP and TILF.

**Discussion:**

This research has provided recommendations to strengthen tourists’ intentions toward local foods by influencing their attitudes, mental beliefs, perceived behavioral control, and risk perception.

## 1. Introduction

Despite the past 2 years, when the world was affected by the outbreak of the COVID-19 virus, it can be argued that tourism has become one of the sectors that impact the world economy. In 2018, the number of international tourists rose to 1.4 billion people (with a 5% increase), and related export income increased by 1.7 trillion dollars (1). This sector has greatly contributed to the growth of GDP and, consequently, the economic development of some countries by boosting the income of host communities (2, 3). So, tourism has motivated the economic development of countries by creating more employment and business (1). Food, as an important part of a tourist’s vacation, may cover one-third of the tourist’s expenses. This issue is one of the main factors in tourists’ decision to travel to a particular destination (4, 5).

Since food is an important factor in tourism (6–9), it is necessary to study the behavior of tourists regarding travel food to use appropriate strategies in the field of tourism management (10). In the past two decades, food tourism has been considered both a research issue in universities and an important issue in the tourism industry (11). It seems that research on the lesser-known dimensions of food tourism, such as tourists’ eating behavior, their intentions and attitudes toward local food, and similar issues, is still attractive.

The study area in the current research is Lorestan province in western Iran. This province is known as an ecotourism destination with the most famous waterfalls, valleys, mountains, natural landscapes, ancient bridges, and historical sites, in addition to a suitable climate. The most famous waterfalls of Lorestan province are Bisheh, Nojyan, Gerit, Vark, and Absefi, which accommodate most of the tourists entering this part of the country. Lorestan province in Iran is known as the land of waterfalls, with Bisheh waterfall, specifically, being known as the “bride of Iran’s waterfalls” (12). Natural, historical, and cultural tourist destinations and, on the other hand, different types of local food in Lorestan province are the reasons for the influx of many tourists to this province. Despite the high potential of the tourism sector and the variety of local food in this province, no research has been conducted on the behavior of tourists toward local food. Therefore, Lorestan province was chosen as the study area in Iran. Research on TILF can guide tourism industry planners to serve local food better. Thus, this study aims to use the extended TPB by adding RP to investigate the TILF in Lorestan province.

Tourism studies are among the research projects assisting with the growing trend of tourism around the world. Considering the subject of the present study, the concepts of tourism, local food, and tourism are explained below.

### 1.1. Food and tourism

Food can be a common language between tourists and hosts because, like any other language, it has the potential to help communicate and share feelings, emotions, and excitement (13). The importance of food in tourism is so high that it can symbolize the culture of a country or a nation (14). These features make it one of the ideal products to be presented as an attraction in tourist destinations (15). One of the other advantages of food when it comes to tourism is its profitability for the host community and, of course, creating pleasure and enjoyment for tourists as guests (16). Eating high-quality foods is a fundamental factor in tourists’ experinece (17). In addition to the importance of tourists’ experiences, food costs also account for a significant part of travel expenses. As a result, it constitutes between a quarter and a third of their total expenditure (18). Food tourism includes activities related to tourists’ nutrition, such as buying and eating local food products or related products. However, we believe that the collection of food from the host farms and the experience of cooking food by tourists together with the host community can also be part of food tourism. Food tourism is more than a typical buying and selling relationship. In addition to the exchange of money and food, it also involves cultural communication, familiarity with the host’s culture, the host’s knowledge of the guest’s culture, and supplier hospitality.

### 1.2. Local food

Local food can be considered as eating a special food in a special tourist destination, which is a different traditional cultural experience due to the uniqueness of the food and the environmental conditions of serving the food (19, 20). According to their national, regional, and individual identities, these specialties can strengthen the image of a destination in the minds of tourists (7, 21, 22). Local food also leads to cultural exchanges between the host community and the visitors, as characteristics such as cultural and environmental authenticity can be recognized in local cuisine (13). When a specific place is chosen as a tourist destination, it is necessary to test the local food of that destination to meet the dietary needs of tourists and their requirements for local experiences (23). Recently, there has been an increase in tourists’ interest in local food in holiday destinations (11). Survey results showed that among tourists visiting the Asia-Pacific region, 90, 87, and 79% seek to experience famous local food, local street food, and unique cultural food, respectively (24). From our point of view, local food can be considered food that has a different taste, ingredients, feel, and experience for tourists compared to the food they have eaten before. New feelings and experiences are achieved both because of the type of food and the special conditions of being in a new environment, which represents a new kind of culture for tourists.

Most of the primary studies on tourist food have been conducted on wine consumption (10). Getz and Brown (25) have investigated the amount and type of wine demand among tourists. Stewart et al. (26) have provided suggestions for tourism development in Canada by examining the fundamental problems related to wine and culinary tourism. The impact of food on tourists’ satisfaction with their travel and experiences at a destination has been confirmed; however, the issue of local food consumption and tourist behavior remains underexplored (4, 27). It is necessary to study food tourism from other angles. One of these interesting aspects is the investigation of the thoughts, attitudes, and behavioral intentions of tourists about these foods. Our better understanding of tourists’ intentions and behaviors will lead to better and more comprehensive food planning in the future.

### 1.3. Theory of planned behavior

TPB (28), an advancement of Fishbein and Ajzen’s rational theory (29), is one of the most famous theories for studying behavior. This theory is an elementary framework for explaining individual behavioral reasons (30). Smarkola (31) argued that in TPB, three variables - perceived behavioral control, mental beliefs, and attitude – have an indirect effect on behavior. The value of TPB lies in considering the relationships between individual, social, and environmental dimensions to explain people’s behavior (32). In this model, behavior is influenced by intention. Other studies show that behavioral intentions are the result of attitude, SN, and PBC (33–35). Due to the general application of this model, extensive studies have been conducted using the TPB on various topics.

**Figure 1:**
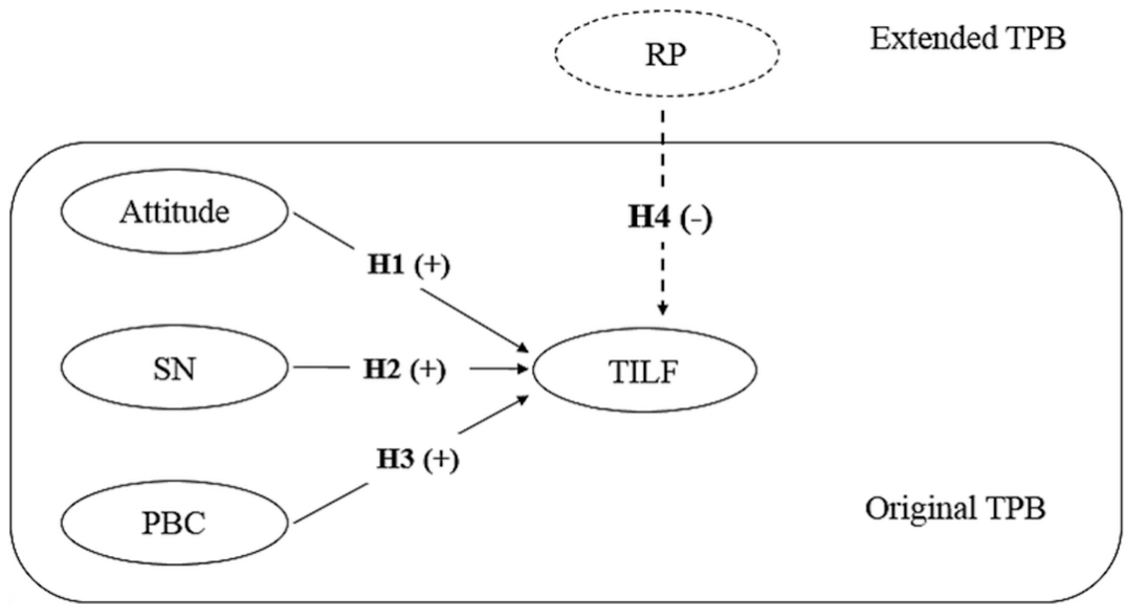
Research framework.

Eating local food is one of the pleasures of traveling. To understand the behavior of tourists in a destination, we must know the hedonic factors (36), because identifying these factors along with understanding the values, attitudes, and beliefs of tourists helps greatly in investigating their behavior (37, 38). To understand and predict the future behavior of tourists in a destination, it is necessary to check the conditions under which local food is used by tourist (36). Therefore, food tourism or food and tourism as a main topic has been the concern of many researchers (20, 39, 40), with many of these authors believing that better decision-making in areas such as marketing and customer relationship management requires a correct understanding of tourists’ eating behaviors and preferences (41) and customer relationship management (42). The TPB is one of the models that is widely used in the analysis of people’s behavior and intentions in different fields. We have also used this model to investigate the intention of tourists toward local food. The existence of the intention variable as well as the important variables of attitude, subjective norms, and perceived behavioral control in this widely used model is one of our basic reasons for using this model. Figure 1 shows the original TPB model. To increase the explanatory power, a risk perception (RP) variable was added to the original model.

#### 1.3.1. Attitude

Attitude indicates the positive or negative appraisal of behavior in a state (43). Attitude is the first TBP variable that affects behavioral intention (44). Any behavior is more likely to occur when a person has a positive attitude toward it (28, 45–49). On the other hand, if a person finds it useful to do something, they do it, and if they find the behavior harmful, they refuse to do it (50). People’s attitude is composed of two related components: emotional and cognitive (51). The emotional part of attitude is related to people’s feelings, and the cognitive component of attitude is related to people’s beliefs (52, 53). Thus, we will also examine attitude in tourists as an essential variable in the TPB.

#### 1.3.2. Subjective norms (SNs)

The second variable that affects people’s intentions is their subjective norms. SNs are influenced by the approval or disapproval of individuals (28, 51, 54). SNs indicate how much each person values the opinions of others in the expression of their behavior (28, 55). People’s behavior is often influenced by what others do, and this influence is greater than that of those with whom they have a close relationship (28, 43, 51). Kim and Chung (56) show that SNs are influenced by the behavior and speech of important people in each person’s life. These include family members, friends, and coworkers. SNs are affected by social norms and perceived social pressure to approve or disapprove of a behavior (57). Eating behaviors and intentions in tourist destinations are usually performed in the presence of others. In other words, tourists usually travel in groups. Tourists’ SNs toward local food consumption may be influenced by their fellow travelers’ behaviors. It is necessary to check the effect of SNs on tourists’ behavioral intentions and behaviors.

#### 1.3.3. Perceived behavioral control (PBC)

The next variable predicting intention in the TPB is PBC. PBC is people’s perception of their abilities for the comfort or difficulty of performing a behavior, which can be the result of successful and unsuccessful experiences in this field (28, 51). On the other hand, PBC refers to a person’s perception of how comfortable or challenging it is to perform a behavior (32, 58). Although it is not always possible to have complete control over opportunities, resources, time, knowledge, and skills, these factors affect a person’s intention to perform a behavior (44). If individuals have more control over their behavior, they are more likely to engage in it (57). In this study, we try to explain the role of PBC in the intention toward local food, and we believe that if people have more control over their behavior, they will more easily express their intention to consume local food.

#### 1.3.4. Intention

In the TPB, a person’s intention to do or not do a behavior directly influences the behavior (43, 51, 59). This variable refers to a conscious plan or decision to act and attempt to behave (28). Intentions are predicted by three key factors: attitude, SN, and PBC (59). The intention of each person has a direct effect on their behavior, and this relationship also applies to the TILF. Therefore, investigating the intention of tourists will be an important issue in predicting their eating behavior. In this research, TILF is the dependent variable.

### 1.4. Extension of TPB

The author argued that new components and relationships will improve this theory (60). The development of the TPB model helps to increase the explanatory power of the model in predicting behavior (28, 61). Other researchers have developed it in different fields and statistical samples to increase the predictive power of this model (44, 62–65). Other studies have also developed this model in the field of tourist eating behavior and intentions (23, 66–70). Here, we attempted to use the extended TPB by adding RP to investigate the TILF towards local food in Iran.

#### 1.4.1. Risk perception

Risk perception refers to a person’s subjective beliefs about the likelihood of a hazard occurring or its severity, timing, and consequences, which may or may not be reasonable (71). RP is a person’s estimate of the probability of a threat and its severity, which usually depends on how people perceive the situation and conditions of the danger (62). This variable is a mental structure. In this specific situation, RP shows whether tourists are afraid of the food they eat or think about how harmful eating this food could be for their health (44). Therefore, RP is an effective variable in people’s decisions when consuming a particular product (71). RP can be considered a strong predictor of intentional behavior (72).

The nature of tourism activities, both during the trip and at the destination, is associated with risk. Therefore, various interpretations of RP have been presented in the tourism and travel literature. The meaning of RP in travel is the awareness of tourists about the uncertainty and potential negative consequences of consuming travel and tourism offers (73, 74). According to Chew and Jahari (75), RP represents the thoughts of tourists about the risks during the trip, which may influence their travel decisions. Food safety, financial, weather, social, physical, psychological, health, service quality, cultural, environmental, and political factors are various categories of perceived risks during travel (73, 76–80). In a few other studies, RP includes physical or health risks (the possibility of catching an infectious disease, epidemics, food safety, and accidents), psychological risks (personal satisfaction from the travel), equipment risks (equipment-related or organizational problems), time risks (the trip could be a waste of time), and social risks (a change in the attitude of friends and relatives toward the tourist due to the trip) are categorized (81–83). Choi et al. (84) have classified the risks related to tourists’ eating habits into health, hygienic, environmental, and socio-psychological risks.

The RP variable has been added to the original TPB model. This is due to the specific characteristics of local food. Also, this is offered only in some special destinations. Tourists may feel threatened when they eat food for the first time in an unknown and new destination. The mentioned cases show the importance of the RP variable in the study of tourists’ intentions. Another reason for adding this variable is the use of other previous studies (23, 84). The higher tourists’ RP of local food, the lower their willingness to consume it. This issue has also been reported in previous studies. We also expect that tourists’ RP will have a negative effect on their intention to consume local food.

### 1.5. Literature review

Sparks (85) investigated wine tourism with the TPB model and concluded that SN and PBC affect tourists’ travel intentions for wine consumption. In addition, television programs and the Internet were important sources of information. Bond et al. (86) argued that, among the TPB variables, attitude has the greatest impact on behavior. The relationship between tourists’ attitudes toward food services/products and their behavioral intentions is usually a direct one (87, 88). The research findings of Karim et al. (89) showed that the relationship between the variables of tourists’ travel satisfaction and their willingness to recommend and revisit Malaysian food with a positive attitude is direct and significant.

Choi et al. (84) investigated the impact of consumers’ RP toward street food and its effect on their attitude toward this type of food and behavioral intention. This study found that perceived risk negatively affects consumer attitude and behavioral intention toward street food. Akkuş and Erdem (90) used the TPB model to investigate tourists’ intention toward food consumption while traveling. They showed that food tourists’ intention can be predicted simply by their attitude and SN values. However, PBC showed no effect on intention. In another study, the key motivations of the participants in a food and beverage exhibition in England were investigated. According to the results, the influence of attitude, SN, and PBC on the subjects’ intention to consume food and drinks in this exhibition was confirmed (66).

Samdin et al. (91) concluded that RP is one of the main concerns of ecotourists when deciding on a destination and affects their decisions during the trip. According to the results, RP affects tourists’ decision-making, so the priority of health and safety information is the strongest predictor. Based on the TPB, Horng et al. (67) investigated the behavior of food festival visitors. The results show that the effect of attitude, SN and PBC on visitors’ intention toward food consumption was significant. The study was most relevant to a research conducted by Zhang et al. (23) in China. This study also examined the factors influencing Chinese domestic tourists’ intention to consume local food based on the extended TPB. Their results show that attitude, SN, and PBC have positive effects on domestic tourists’ behavioral intention to consume local food. In addition, RP has a negative relationship with behavioral intention. The study by Vesci and Botti (70), which used the developed TPB, evaluated the main characteristics of local Italian culinary festivals and their effects on the attitude of visitors. The results show that food and beverage quality, staff service, and information strongly determine the participants’ attitude toward local festivals and their intention to revisit them.

Memon et al. (92) investigated the factors influencing the intention of international students to consume local food in Malaysia. According to the results of this research, the three variables of attitude, subject norms, and PBC significantly affect students’ behavior toward Malay food consumption. SN, as the strongest explainer, had the highest impact on students’ behavior.

Prapasawasdi et al. (93) revealed that attitude, SN, perceived value, and expectation factors of Thai tourists had a positive effect on how they perceived local food information.

By adding the variable of health consciousness to the TPB, Shin et al. (68) investigated the intention of consumers toward government-branded food products. The results indicate that consumers’ attitude, SN, and PBC significantly predict their intention to consume local food. However, the effect of health consciousness on consumers’ intention was not found. In research by Su et al. (69), the TPB model was used by adding two factors - food travel motivation and destination food perspective - to understand the behavioral goals of diners toward food tourism. The findings empirically show the relationship between motivation, attitude, and behavioral intention. It supports food tourism. Attitude mediates the relationship between travel food and food consumption motivation at the destination with behavioral intention. Hamid and Azhar (94) revealed that consumers’ behavioral intention to order food and beverages during COVID-19 was affected by three variables, attitude, SN, and trust, and that the effect of PBC on behavior was insignificant. Dedeoğlu et al. (95) showed in their study how international tourists’ local food consumption intentions are affected by the main variables of the theory of planned behavior (TPB). According to the results, attitude toward local food and perceived behavioral control have a positive and significant effect on the intention to consume local food.

The attitude of tourists toward local food has been investigated using other models. Another study has shown that the attitude of tourists toward local food is influenced by taste and quality, health, price, emotional value, and food prestige value (27).

### 1.6. Research gap

The literature review related to the topic of this research shows that only two studies by Dedeoğlu et al. (95) and the study by Zhang et al. (23). The rest of the studies are in fields close to this topic. For example, examining the relationship between the variables of tourists’ travel satisfaction and their greater willingness to recommend and visit again with a positive attitude toward local food is one of these cases. Some of these studies have focused on the intention to consume government-branded food, street food, or beverages. Some other studies have been conducted in statistically different communities than tourists, such as international students in the host country. A number of other studies have been conducted to investigate the intention or attitude of visitors to festivals and food exhibitions.

The attractiveness of local food and the intention of tourists to consume these products are so great that the limited number of studies mentioned in this field does not seem sufficient. This shows that we are still facing a lack of comprehensive research in the field of scientific literature related to this issue. The current research is an attempt to fill a part of the deep gap that exists regarding local food.

### 1.7. Hypotheses of the original and extended TPB models

We have proposed five hypotheses *H1*–*H3*. These hypotheses are presented based on the variables of the original TPB model and the relationships among these variables. Also, based on the information provided about the RP of the extended TPB model, the *H4* hypothesis is proposed (Figure 1). The hypotheses are formulated as follows:

*H1*: Tourists’ attitude has a positive and significant effect on TILF.

*H2*: Tourists’ SN has a positive and significant effect on their TILF.

*H3*: Tourists’ PBC has a positive and significant effect on their TILF.

*H4*: Tourists’ RP has a negative and significant effect on their TILF.

## 2. Methodology

### 2.1. Study area

**Figure 2:**
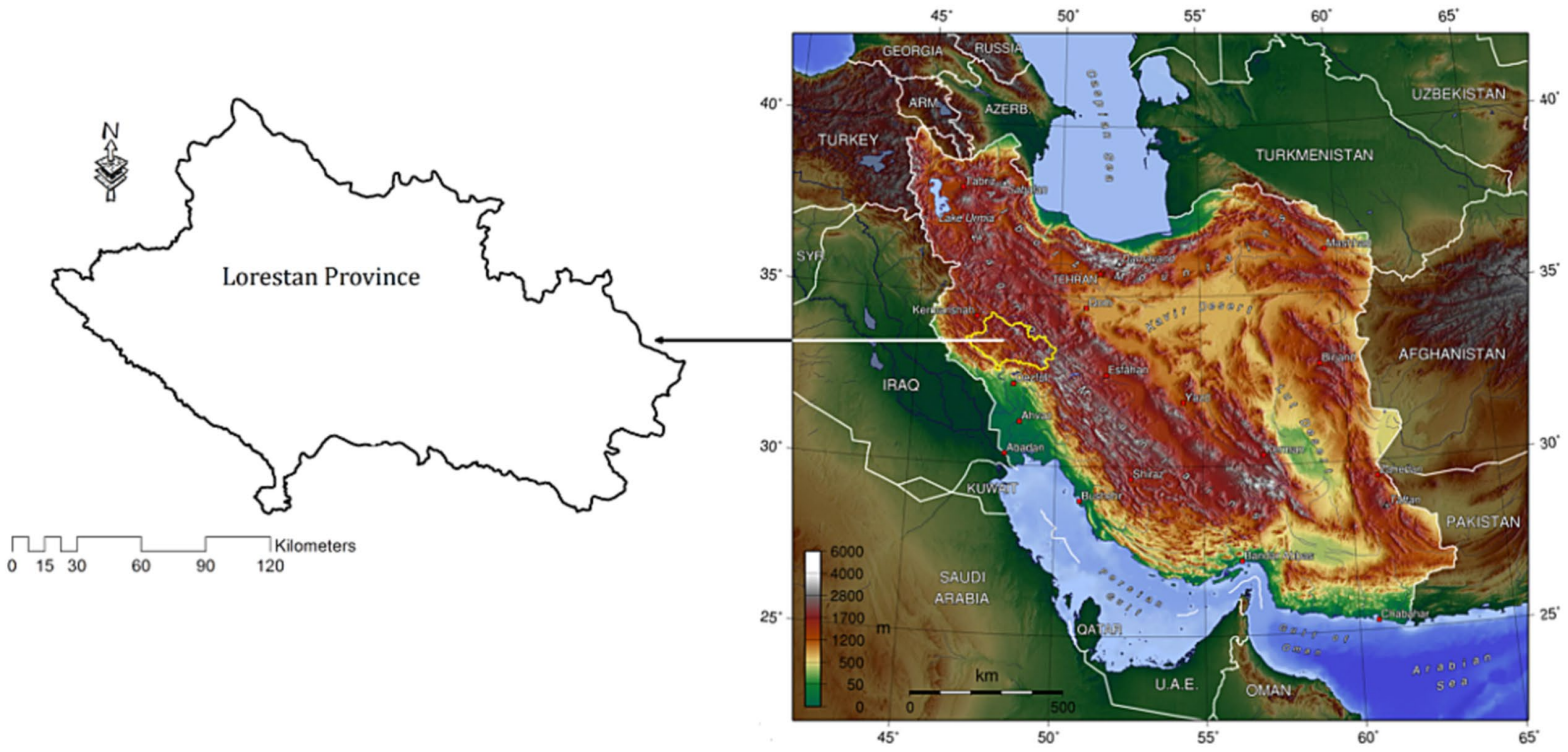
Survey locations for the study (12).

The study area in the current research is Lorestan, a province in western Iran. The research was conducted in 2021. Lorestan province is located between the latitudes of 32° 30′ and 48°1′ N and the longitudes of 55°17′ and 61° 15′ E. The surface of this province is 28,064 km^2^ (44). Figure 2 displays its location.

### 2.2. Samples, sample size determination, and sampling

The samples of this research include tourists who have traveled to areas of Lorestan province where local food is served. For better distribution of samples in terms of time, the middle month of each season in 2021 was selected. Samples were therefore taken in February, May, August, and November. According to the tourist office of Lorestan province, the number of these tourists was estimated to be 110,000. Using Cochran’s formula, the number was estimated at 300. However, 313 questionnaires were completed for greater accuracy. For better spatial distribution of the samples, five cities from the north, south, center, west, and east were selected from 10 cities in Lorestan province. Two local food restaurants were selected from each city. Then, approximately 30 tourists were selected from each restaurant during the mentioned months. Data were collected through a questionnaire and face-to-face interviews with tourists.

### 2.3. Measurements

A survey questionnaire was designed that consisted of the following two parts: (a) tourist demographics, (b) questions related to measuring the variables of attitude (4 items), SN (3 items), PBC (3 items), intention (3 items), and RP (3 items). All items were measured with a 5-point Likert scale (96).

### 2.4. Reliability and validity of the questionnaire

We asked a team of tourism office employees and tour guides to identify the flaws in the questionnaire. Therefore, before entering the interview stage with the samples, the validity of the questionnaire was confirmed by this team. The reliability of the scales used in the questionnaire was also confirmed by the value of Cronbach’s alpha coefficient, which was above 0.7. The suitability of the collected data for structural equation modeling (SEM) was done using confirmatory factor analysis. Table 1 shows that the goodness of fit evaluation indices are sufficient in both measurement models.

**Table 1:** Summary of goodness of fit indices for the measurement model.

Fit index	SRMR	D-G1	D-G2	NFI	RMS-Theta
Suggested value	<0.1	>0.05	>0.05	>0.90	≤0.12
Original TPB	0.08	0.317	0.378	0.94	0.07
Extended TPB	0.08	0.319	0.399	0.95	0.06

Table 2 shows the diagnostic validity results. The mean of the variance extracted for the research structures (0.81 < AVE < 0.87) was greater than the correlation between them (0.52 < *r* < 0.78). This result showed that the diagnostic validity of the structures in the proposed research model was confirmed.

**Table 2:** Correlations with the square roots of the AVE.

Constructs	1	2	3	4	5
1. Attitude	0.87^a^				
2. PBC	0.63^**^	0.85^a^			
3. RP	0.78^**^	0.78^**^	0.81^a^		
4. SN	0.52^**^	0.66^**^	0.75^**^	0.87^a^	
5. TILF	0.79^**^	0.77^**^	0.76^**^	0.68^**^	0.80^a^

^a^Square root of the AVE estimate. **Correlation is significant at the < 0.01 level.

### 2.5. Data analysis

The collected data were analyzed with SPSS20 and Smart PlS software. In the descriptive statistics, the demographic and travel characteristics of tourists are described using statistics such as frequency and percentage. SEM is one of the statistical techniques suitable for investigating the relationship between several variables in a model (97). We also used this method for hypothesis testing on both models. This analysis was done using PLS software. The main reason for using SEM is that this is a complete method to test the hypothesis of a study. The second reason for using this method is that we can report the measurement error (98). In this method, the relationship between hidden and observed variables, and also among some hidden factors are investigated by determining the intensity of their internal relationships in the set of equations (99).

## 3. Results

### 3.1. Tourist demographics

The demographics of tourists are presented in Table 3. Women are 12.4% more than men. A little more than a quarter (26.5%) of tourists are between 36 and 45 years old. Only 17.9% are over 55 years old. Married tourists travel more than single tourists to this area (66.1 to 33.9%). Approximately 60% of tourists have a bachelor’s degree or higher. Only 3.5% are illiterate. Compared to other occupations, private sector employers are more receptive to the study area as a tourist destination (42.5%). The monthly household income of approximately 47.3% of tourists is between US $300 and $500.

**Table 3:** Tourist demographics (*N* = 313).

Tourist demographics	Frequency	Percentage
*Gender*		
**Women**	**176**	**56.2**
Men	137	43.8
*Age*		
Less than 25	44	14.1
25–35	65	20.8
36–45	83	26.5
46–55	65	20.8
55 and over	56	17.9
*Marital status*		
Single	106	33.9
**Married**	207	66.1
*Education*		
Illiterate	11	3.5
Primary school	27	8.6
Secondary school	31	9.9
High school	56	17.9
Associate degree	71	22.7
**Bachelor’s degree or higher**	117	37.4
*Occupation*		
Civil servant	109	34.8
**Private sector employee**	133	42.5
Unemployed	71	22.7
*Monthly household income (6,000,000 Toman = US $300)*		
Less than 6,000,000	116	37
6,000,001–10,000,000	148	47.3
More than 10,000,000	49	15.7

#### 3.1.1. Measurement model

The appropriateness of the two original and extended models in this study was validated by confirmatory factor analysis (CFA). The results in Table 4 show that both models have an appropriate fit. The t-values for all the standardized factor loadings (λ) in all the selected indicators show that the relationships between all indicators and each related construct are significant (with a 0.01 level of error and *p* < 0.01). Based on these results, the selected indicators were confirmed in each of the measurement models. In other words, appropriate indicators were selected to measure the latent variables of both models. Table 4 shows that the values of AVE, CR, and α are greater than 0.5, 0.6, and 0.7, respectively. These results confirm the convergent validity and reliability of all the latent variables in the proposed model (Table 4). The comparison of all models’ goodness of fit indices with respect to the recommended value also indicates the appropriate goodness of fit of both models (see Table 1: SRMR <0.10, D_G1 > 0.05, D_G2 > 0.05, NFI > 0.90, RMS_Theta ≤0.12).

**Table 4:** The goodness of fit of the models.

Constructs	Measurement item	Original TPB			Extended TPB		
		*λ*	*t*	Reliability and Validity statistics	*λ*	*t*	Reliability and Validity statistics
Attitude	Attit_1_	0.856	23.63	AVE: 0.762 CR: 0.928 α: 0.896	0.857	23.94	AVE: 0.762 CR: 0.928 α: 0.896
	Attit_2_	0.877	28.68		0.877	29.12	
	Attit_3_	0.886	42.95		0.886	44.02	
	Attit_4_	0.873	40.75		0.873	41.08	
SN (Subjective Norm)	SN_1_	0.846	27.03	AVE: 0.773 CR: 0.911 α: 0.853	0.847	27.47	AVE: 0.773 CR: 0.911 α: 0.853
	SN_2_	0.889	50.59		0.889	50.05	
	SN_3_	0.900	59.54		0.900	58.25	
PBC (Perceived Behavioral Control)	PBC_1_	0.859	42.85	AVE: 0.737 CR: 0.894 α: 0.824	0.859	41.79	AVE: 0.737 CR: 0.894 α: 0.824
	PBC_2_	0.889	46.11		0.889	45.29	
	PBC_3_	0.827	25.04		0.827	25.18	
TILF Intention	Inten_1_	0.799	26.61	AVE: 0.649 CR: 0.847 α: 0.730	0.795	29.54	AVE: 0.649 CR: 0.847 α: 0.730
	Inten_2_	0.821	19.93		0.822	18.90	
	Inten_3_	0.797	31.62		0.799	32.37	
RP (Risk Perception)	RP_1_	–	–	–	0.804	24.95	AVE: 0.661 CR: 0.854 α: 0.746
	RP_2_	–	–		0.794	17.18	
	RP_3_	–	–		0.839	31.10	

#### 3.1.2. Structural model

The results related to the validation of both models have been presented in paragraph 3.4.1. After validating the results of the measurement models, the results of the original and extended TPB models presented below.

#### 3.1.3. Original TPB model

**Figure 3:**
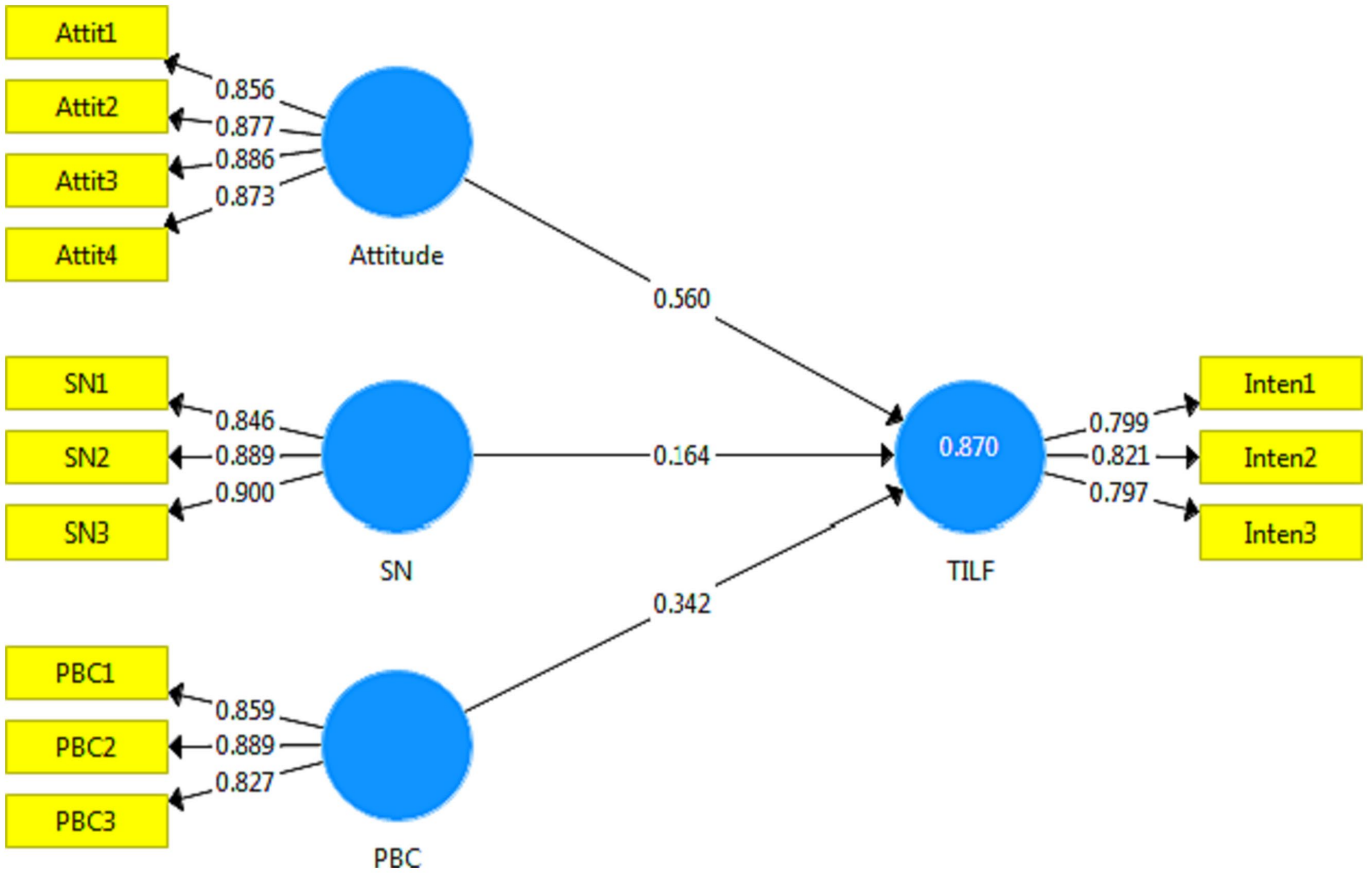
Original TPB structural model with standardized path coefficients.

The results of Figure 3 show that the variables of attitude, SN, and PBC can explain 87% of the variance of c TILF (Figure 3). All three variables are positively and significantly correlated with this variable. These variables, in order of importance, are attitude, SN, and PBC are important in order. Based on these results, the hypotheses of the original model (*H1*–*H3*) are confirmed (Table 5).

**Table 5:** Hypothesis research results and structural models.

Hypothesis	Original TPB			Extended TPB		
	*γ*	*t*	Result	*γ*	*t*	Result
*H1*: Attitude → TILF	0.560	15.567	Confirmed	0.648	13.874	Confirmed
*H2*: SN → TILF	0.164	5.525	Confirmed	0.250	5.648	Confirmed
*H3*: PBC → TILF	0.342	11.711	Confirmed	0.443	9.243	Confirmed
*H4*: PBC → TILF	–	–	–	−0.250	2.766	Confirmed

#### 3.1.4. Extended model

**Figure 4:**
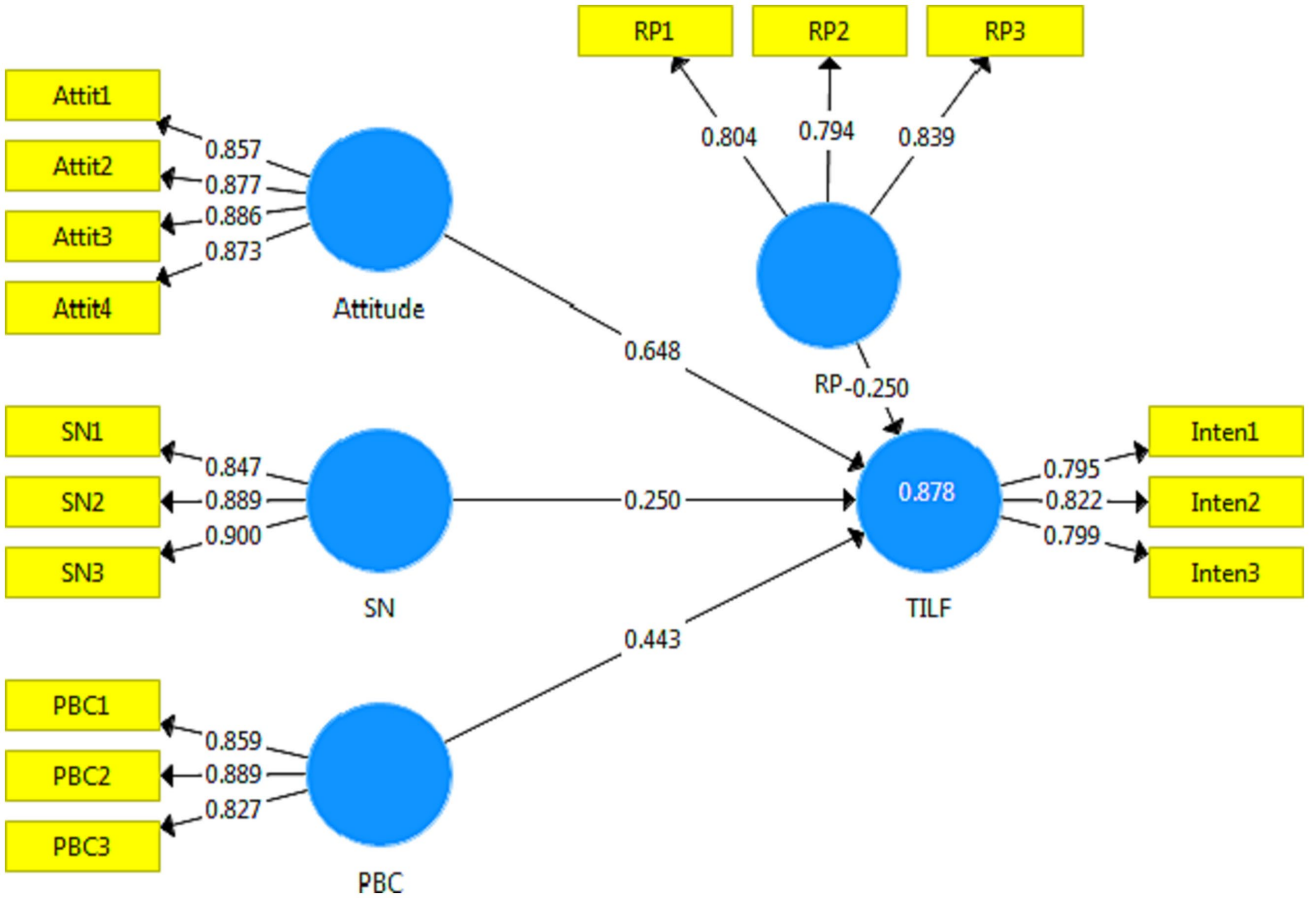
Extended TPB structural model with standardized path coefficients.

The results of Figure 4 show that in the developed model, 87.8% of the variance was explained by TILF. The effect of RP on TEF is significant. The value of the *T*-Values for this variable is more than 1.96 (98). The value of the coefficient between the two variables is also negative. The results show that there is a negative and significant relationship between this variable and the TILF (Table 5; Figure 4).

## 4. Discussion

Many researchers investigating people’s behavior on different topics use TPB. The basic reason is the ability of the theory elements to predict tourists’ intention. Therefore, the original TPB was used to explain TILF. In addition, we tried to develop the explanatory power of the original model by adding the RP variable.

The SEM analysis revealed that attitude, SN, PBC, and RP in the extended model explained 87.8% of the variance of TILF. The findings indicated that these variables explained tourists’ intention. All three variables in the original model were positively and significantly correlated with TILF. The extended model also showed that the RP variable had a negative and significant effect on TILF. Therefore, a new TPB model for the investigation of tourists’ intention was proposed. The results of the hypothesis testing are discussed in the following order:

*H1*: Tourists’ attitude toward local food has a positive and significant effect on TILF. As the positive effect of the attitude variable on behavioral intention has been reported in other studies (23, 67, 87, 88), the direct impact of this variable on TILF is confirmed in our research. Bond et al. (86) believe that, among the TPB variables, attitude has the greatest impact on behavior. Attitude is a vital explanatory variable in direct intention analysis. This is because the positive or negative attitude of each person toward a topic can be an important intellectual basis for deciding whether or not to choose that topic. This issue may be more apparent in the field of travel food choices for tourists. In addition to having an effect on the health of tourists during the trip, travel food will lead to the creation of an experience and imagination in the minds of tourists. Karim et al. (89) showed that a positive attitude toward travel food increases tourists’ travel satisfaction and their willingness to revisit the destination.

The results of this research show that people’s attitude has a direct effect on their behavioral intention. Educational programs in some media, including TV and Radio can be helpful to change the attitude of tourists to local food in the long term. People’s attitude is changed by making educational programs about local food and broadcasting them in public media such as radio and television in the long term. Some measures to change the attitude of tourists in the short term can be undertaken when tourists arrive in the studied province, such as distributing brochures about local foods in Lorestan province, reliable and healthy stores selling these, etc. One of the fundamental suggestions of this research is the establishment of a food and tourism television channel at the national level that only presents tourist sites, rituals, and customs of Iranian ethnic groups and their regional foods. The study by Vesci and Botti (70) also showed that information strongly determines the participants’ attitude toward local food festivals and their intention to revisit a destination.

*H2*: Tourists’ SN toward local food has a positive and significant effect on TILF, which is in agreement with the findings of Duarte Alonso et al. (66) and Zhang et al. (23). Memon et al. (92) also reported that the SN variable, as the strongest explainer, has the greatest impact on the behavior of international students to consume local food in Malaysia. Family, friends, travel companions, and influential people in each person’s life determine the strengths and weaknesses of their SNs. Usually, the parents in each family decide the type of food to be consumed on the trip. Improving the parents’ beliefs about local food will have a positive effect on the SNs of their family members. According to Sparks (85), television programs and the Internet are important sources of information to strengthen tourists’ SN. In addition, it is possible to improve tourists’ SN to consume local foods in Lorestan province through a number of activities. These include establishing restaurants that serve local food and drinks, supporting restaurants that are active in selling traditional food, and creating a permanent booth in all tourism exhibitions that introduces local food and local souvenirs from Lorestan province.

*H3*: Tourists’ PBC toward local food has a positive and significant effect on TILF. The effect of PBC on tourists’ intention to consume local food has been confirmed in most studies (23, 66–68, 95), and not confirmed in some studies (90, 94).

PBC shows the intensity and weakness of people’s control over their intentions and behavior, and the extent to which people can control their behavior toward a specific issue. This variable indicates that a person has sufficient authority to perform or not perform a behavior. The results of this research show that the more people have the choice of their travel food during the trip, the greater their desire to consume local food. This work also shows that the local cuisine of Lorestan province attracts tourists. An important point is the influence of parents on children’s food choices. Usually, in societies like Iran, parents decide the food for the whole family. According to this result, it is recommended to promote the culture of “freely ordering food by each person” among tourists through mass education and media. Another suggestion is that by diversifying the list of local and new food in the restaurants of Lorestan province, the possibility of ordering food is provided to all family members of tourists and travelers. In this case, people can decide which food to choose on their trip. This issue requires training of restaurant owners, hoteliers, and managers of local food distribution centers by the Cultural Heritage and Tourism Organization of Lorestan Province.

*H4*: Tourists’ RP toward local food has a negative and significant effect on TILF. As mentioned in Section 1.4.1, RP refers to people’s subjective beliefs about the likelihood of a hazard occurring or its severity, timing, and consequences. This variable deals with the level of risk felt by tourists when eating travel food. This unpleasant feeling is caused by their fear of getting sick, the poor taste and quality of food, or the incompatibility of the local food with their food taste. If tourists feel that eating food is dangerous to their health, they will probably refrain from eating it. Even their desire to choose that food will decrease. Samdin et al. (91) argued that health and safety are the strongest Therefore, the results of the present study are not far from reality. Other researchers also confirm this conclusion in their research about local food and other edibles (23, 84). The main recommendation of the current research is to ensure that the local food is healthy and to reassure tourists of its good quality in tourist destinations. It is very important to implement this because many tourists may be experiencing local food for the first time, and the healthiness of these products is unknown to them. Tourism organizations and the Ministry of Health must supervise the preparation of local food in tourist destinations. It is also necessary to provide information about what is offered, such as the presence of raw ingredients, its calorie content, whether it is organic or not, cooking methods, and so on, through brochures or postings on restaurants’ websites. Training restaurant owners about local food and, especially, their cooks to prepare healthy local food can be useful in this field.

## 5. Conclusion

Unlike in the past few decades, tourist food is no longer a trivial and unimportant issue. This has become a fundamental priority for tourists and tourism service providers. For example, a large part of a tourist’s travel expenses revolves around buying food, and a large part of the income of tourism service providers is obtained from the sale of food. The importance of tourist food and the attempt to answer its ambiguities make clear the need for scientific and comprehensive research in this direction. Also, local foods have become more important for tourists.

In addition, local food has a cultural value for tourists in the study location. By eating local food, tourists experience different tastes and get to know the food culture of the host community. The present research is one of the attempts to investigate the intentions of tourists toward local food. Considering the widespread use of the TPB in analyzing the behaviors and intentions of people in different fields, we also used this model to study the intentions of tourists toward consuming local food. We added the RP variable to the model, considering its importance to the issue of food safety. Few studies have been conducted to investigate tourists’ intention toward local food using the TPB and adding the RP variable. This can be considered one of the innovations of the present study. This was the first research, considering the lack of studies in this area in Iran.

Our recommendation regarding future research prospects is that other researchers conduct studies on tourist behavior using other models, such as the Health Belief Model (HBM) and the Kim and Cho model. Other models may provide different results due to different variables. Examining the intentions of tourists to eat local food by considering their individual variables, such as gender, age, education, income level, and eating habits, is also one of the other recommendations for future research.

It is necessary to mention the limitations of this research. Filling out the questionnaires by tourist can disturb them during eating food. Doing so is not pleasant for a person who wants to enjoy their food. We tried to do this while the tourists were waiting for their ordered food to be ready. For solving this problem, we waited to finish eating food. The second limitation was also related to the collection of the questionnaires. The majority of restaurateurs did not want a researcher to be present in their establishments.

The last limitation was the lack of studies that have exactly investigated tourists’ preferences for local food with the TPB model. To overcome this limitation, we had to use some relatively related studies that are indicated in the literature review section.

## Data availability statement

The raw data supporting the conclusions of this article will be made available by the authors, without undue reservation.

## Ethics statement

Written informed consent was obtained from the individual(s), and minor(s)’ legal guardian/next of kin, for the publication of any potentially identifiable images or data included in this article.

## Author contributions

MM: conceptualization and data curation. MR: methodology, software, and writing – original draft preparation. SG: supervision and validation. All authors contributed to the article and approved the submitted version.

## References

[1] UNWTO. International tourism highlights. Madrid: UNWTO (2019).

[2] Azam M Alam MM Hafeez MH. Effect of tourism on environmental pollution: further evidence from Malaysia, Singapore and Thailand. J Clean Prod. (2018) 190:330–8. doi: 10.1016/j.jclepro.2018.04.168

[3] Joshi O Poudyal NC Larson LR. The influence of sociopolitical, natural, and cultural factors on international tourism growth: a cross-country panel analysis. Environ Dev Sustain. (2017) 19:825–38. doi: 10.1007/s10668-016-9767-x

[4] Cheng Q Huang R. Is food tourism important to Chongqing (China). J Vacat Mark. (2015) 1:225–36. doi: 10.1177/1356766715589427

[5] Im HH Kim SS Elliot S Han H. Conceptualizing destination brand equity dimensions from a consumer-based brand equity perspective. J Travel Tour Mark. (2012) 29:385–403. doi: 10.1080/10548408.2012.674884

[6] Du Rand GE Heath E. Towards a framework for food tourism as an element of destination marketing. Curr Issue Tour. (2006) 9:206–34. doi: 10.2167/cit/226.0

[7] Henderson JC. Food tourism reviewed. Br Food J. (2009) 111:317–26. doi: 10.1108/00070700910951470

[8] Robinson RN Getz D. Profiling potential food tourists: an Australian study. Br Food J. (2014) 116:690–706. doi: 10.1108/BFJ-02-2012-0030

[9] Tsai C-TS Wang Y-C. Experiential value in branding food tourism. J Destin Mark Manag. (2017) 6:56–65. doi: 10.1016/j.jdmm.2016.02.003

[10] Vu HQ Li G Law R Zhang Y. Exploring tourist dining preferences based on restaurant reviews. J Travel Res. (2019) 58:149–67. doi: 10.1177/0047287517744672

[11] Okumus B. Food tourism research: a perspective article. Tour Rev. (2020) 76:38–42. doi: 10.1108/TR-11-2019-0450

[12] Asadpourian Z Rahimian M Gholamrezai S. SWOT-AHP-TOWS analysis for sustainable ecotourism development in the best area in Lorestan Province. Iran Soc Indicat Res. (2020) 152:289–315. doi: 10.1007/s11205-020-02438-0

[13] Sidali KL Spiller A Schulze B. Food, agri-culture and tourism: Linking local gastronomy and rural tourism: Interdisciplinary perspectives Springer Science & Business Media (2011).

[14] Kim S Iwashita C. Cooking identity and food tourism: the case of Japanese udon noodles. Tour Recreat Res. (2016) 41:89–100. doi: 10.1080/02508281.2016.1111976

[15] Moginon D. F. See T. P. Saad M. (2012). “Indigenous food and destination marketing,” in Current Issues in Hospitality and Tourism Research and Innovations-Proceedings of the International Hospitality and Tourism Conference, IHTC. 355–358.

[16] Sims R. Food, place and authenticity: local food and the sustainable tourism experience. J Sustain Tour. (2009) 17:321–36. doi: 10.1080/09669580802359293

[17] Pizam A Jeong G-H Reichel A van Boemmel H Lusson JM Steynberg L. The relationship between risk-taking, sensation-seeking, and the tourist behavior of young adults: a cross-cultural study. J Travel Res. (2004) 42:251–60. doi: 10.1177/0047287503258837

[18] Correia A Moital M Da Costa CF Peres R. The determinants of gastronomic tourists’ satisfaction: a second-order factor analysis. J foodserv. (2008) 19:164–76. doi: 10.1111/j.1745-4506.2008.00097.x

[19] Hall CM Mitchell R Sharples L. Consuming placesthe role of food, wine and tourism in regional development: the role of food, wine and tourism in regional development. In: Food tourism around the world: Routledge (2004). 25–59.

[20] Kim YG Eves A. Construction and validation of a scale to measure tourist motivation to consume local food. Tour Manag. (2012) 33:1458–67. doi: 10.1016/j.tourman.2012.01.015

[21] Bessière J. Local development and heritage: traditional food and cuisine as tourist attractions in rural areas. Sociol Rural. (1998) 38:21–34. doi: 10.1111/1467-9523.00061

[22] Chang RC Kivela J Mak AH. Food preferences of Chinese tourists. Ann Tour Res. (2010) 37:989–1011. doi: 10.1016/j.annals.2010.03.007

[23] Zhang H Li L Yang Y Zhang J. Why do domestic tourists choose to consume local food? The differential and non-monotonic moderating effects of subjective knowledge. J Destin Mark Manag. (2018) 10:68–77. doi: 10.1016/j.jdmm.2018.06.001

[24] The Way to Singapo (2014). The way to Singaporean leisure travelers’ hearts is through their stomachs, The Way to Singapore.

[25] Getz D Brown G. Critical success factors for wine tourism regions: a demand analysis. Tour Manag. (2006). doi: 10.1016/j.tourman.2004.08.002

[26] Stewart JW Bramble L Ziraldo D. Key challenges in wine and culinary tourism with practical recommendations. Int J Contemp Hosp Manag. (2008) 20:303–12. doi: 10.1108/09596110810866118

[27] Rousta A Jamshidi D. Food tourism value: investigating the factors that influence tourists to revisit. J Vacat Mark. (2020) 26:73–95. doi: 10.1177/1356766719858649

[28] Ajzen I. The theory of planned behavior. Organ Behav Hum Decis Process. (1991) 50:179–211. doi: 10.1016/0749-5978(91)90020-T

[29] Koen J Klehe U-C Van Vianen AE. Employability among the long-term unemployed: a futile quest or worth the effort? J Vocat Behav. (2013) 82:37–48. doi: 10.1016/j.jvb.2012.11.001

[30] Lubran M. (2010). Factors influencing maryland farmers’on-farm processing license application behavior. University of Maryland, College Park.

[31] Smarkola C. Efficacy of a planned behavior model: beliefs that contribute to computer usage intentions of student teachers and experienced teachers. Comput Hum Behav. (2008) 24:1196–215. doi: 10.1016/j.chb.2007.04.005

[32] Savari M Gharechaee H. Utilizing the theory of planned behavior to predict Iranian farmers’ intention for safe use of chemical fertilizers. J Clean Prod. (2020) 263:121512. doi: 10.1016/j.jclepro.2020.121512

[33] Brown K. (1998). Social cognitive theory [Online] Available at: http://hsc.usf.edu/~kmbrown.Social_Cognitive_Theory_Overview.htm

[34] Glanz K Rimer BK Viswanath K. Health behavior and health education: Theory, research, and practice. Jossey-Bass: John Wiley & Sons (2008).

[35] Rhodes RE Macdonald HM McKay HA. Predicting physical activity intention and behaviour among children in a longitudinal sample. Soc Sci Med. (2006) 62:3146–56. doi: 10.1016/j.socscimed.2005.11.051, PMID: 16406632

[36] Choe JYJ Kim SS. Effects of tourists’ local food consumption value on attitude, food destination image, and behavioral intention. Int J Hosp Manag. (2018) 71:1–10. doi: 10.1016/j.ijhm.2017.11.007

[37] Kilbourne W Pickett G. How materialism affects environmental beliefs, concern, and environmentally responsible behavior. Journal of Business Research. (2008) 61:885–893.

[38] Gonçalves J Mateus R Silvestre J. D. Roders A. P. Bragança L. Attitudes matter: Measuring the intention-behaviour gap in built heritage conservation. Sustainable Cities and Society. (2008) 70:102913.

[39] Ellis A Park E Kim S Yeoman I. What is food tourism? Tour Manag. (2018) 68:250–63. doi: 10.1016/j.tourman.2018.03.025

[40] Kim YG Eves A Scarles C. Building a model of local food consumption on trips and holidays: a grounded theory approach. Int J Hosp Manag. (2009) 28:423–31. doi: 10.1016/j.ijhm.2008.11.005

[41] Min K-H Lee TJ. Customer satisfaction with Korean restaurants in Australia and their role as ambassadors for tourism marketing. J Travel Tour Mark. (2014) 31:493–506. doi: 10.1080/10548408.2013.877412

[42] Lee A Lambert CU Law R. Customer preferences for social value over economic value in restaurants. Asia Pac J Tour Res. (2012) 17:473–88. doi: 10.1080/10941665.2011.627350

[43] Kaiser FG Scheuthle H. Two challenges to a moral extension of the theory of planned behavior: moral norms and just world beliefs in conservationism. Personal Individ Differ. (2003) 35:1033–48. doi: 10.1016/S0191-8869(02)00316-1

[44] Ahmmadi P Rahimian M Movahed RG. Theory of planned behavior to predict consumer behavior in using products irrigated with purified wastewater in Iran consumer. J Clean Prod. (2021) 296:126359. doi: 10.1016/j.jclepro.2021.126359

[45] Ajzen I. From intentions to actions: a theory of planned behavior In: Kuhl J Beckmann J, editors. Action control. Berlin, Heidelberg: Springer (1985). 11–39.

[46] Davis LE Ajzen I Saunders J Williams T. The decision of African American students to complete high school: an application of the theory of planned behavior. J Educ Psychol. (2002) 94:810–9. doi: 10.1037/0022-0663.94.4.810

[47] Läpple D Kelley H. Understanding the uptake of organic farming: accounting for heterogeneities among Irish farmers. Ecol Econ. (2013) 88:11–9. doi: 10.1016/j.ecolecon.2012.12.025

[48] Massoud MA Terkawi M Nakkash R. Water reuse as an incentive to promote sustainable agriculture in Lebanon: stakeholders’ perspectives. Integr Environ Assess Manag. (2019) 15:412–21. doi: 10.1002/ieam.4131, PMID: 30690841

[49] Wauters E Bielders C Poesen J Govers G Mathijs E. Adoption of soil conservation practices in Belgium: an examination of the theory of planned behaviour in the agri-environmental domain. Land Use Policy. (2010) 27:86–94. doi: 10.1016/j.landusepol.2009.02.009

[50] Hrubes D Ajzen I Daigle J. Predicting hunting intentions and behavior: an application of the theory of planned behavior. Leis Sci. (2001) 23:165–78. doi: 10.1080/014904001316896855

[51] De Bruijn G-J. Understanding college students’ fruit consumption. Integrating habit strength in the theory of planned behaviour. Appetite. (2010) 54:16–22. doi: 10.1016/j.appet.2009.08.007, PMID: 19712718

[52] Arvola A Vassallo M Dean M Lampila P Saba A Lähteenmäki L. Predicting intentions to purchase organic food: the role of affective and moral attitudes in the theory of planned behaviour. Appetite. (2008) 50:443–54. doi: 10.1016/j.appet.2007.09.010, PMID: 18036702

[53] Farley SD Stasson MF. Relative influences of affect and cognition on behavior: are feelings more related to blood donation intentions? Exp Psychol. (2003) 50:55–62. doi: 10.1027//1618-3169.50.1.55 12629961

[54] Chen M-F Tung P-J. Developing an extended theory of planned behavior model to predict consumers’ intention to visit green hotels. Int J Hosp Manag. (2014) 36:221–30. doi: 10.1016/j.ijhm.2013.09.006

[55] Ajzen I. (2006). Constructing a theory of planned behavior questionnaire: conceptual and methodological considerations. Available at: http://www.people.umass.edu/aizen/pdf/tpb.measurement.pdf

[56] Kim HY Chung JE. Consumer purchase intention for organic personal care products. J Consum Mark. (2011) 28:40–47.

[57] Goh E Ritchie B Wang J. Non-compliance in national parks: an extension of the theory of planned behaviour model with pro-environmental values. Tour Manag. (2017) 59:123–7. doi: 10.1016/j.tourman.2016.07.004

[58] De Leeuw A Valois P Ajzen I Schmidt P. Using the theory of planned behavior to identify key beliefs underlying pro-environmental behavior in high-school students: implications for educational interventions. J Environ Psychol. (2015) 42:128–38. doi: 10.1016/j.jenvp.2015.03.005

[59] Russell S Fielding K. Water demand management research: a psychological perspective. Water Resour Res. (2010) 46. doi: 10.1029/2009WR008408

[60] Ajzen I. Attitudes, personality, and behavior. UK: McGraw-Hill Education (2005).

[61] Perugini M Bagozzi RP. The role of desires and anticipated emotions in goal-directed behaviours: broadening and deepening the theory of planned behaviour. Br J Soc Psychol. (2001) 40:79–98. doi: 10.1348/014466601164704, PMID: 11329835

[62] Azadi Y Yazdanpanah M Mahmoudi H. Understanding smallholder farmers’ adaptation behaviors through climate change beliefs, risk perception, trust, and psychological distance: evidence from wheat growers in Iran. J Environ Manag. (2019) 250:109456. doi: 10.1016/j.jenvman.2019.109456, PMID: 31513997

[63] Meng B Choi K. Extending the theory of planned behaviour: testing the effects of authentic perception and environmental concerns on the slow-tourist decision-making process. Curr Issue Tour. (2016) 19:528–44. doi: 10.1080/13683500.2015.1020773

[64] Sultan MT Sharmin F Badulescu A Stiubea E Xue K. Travelers’ responsible environmental behavior towards sustainable coastal tourism: an empirical investigation on social media user-generated content. Sustainability. (2021) 13:56. doi: 10.3390/su13010056

[65] Wang C Zhang J Yu P Hu H. The theory of planned behavior as a model for understanding tourists’ responsible environmental behaviors: the moderating role of environmental interpretations. J Clean Prod. (2018) 194:425–34. doi: 10.1016/j.jclepro.2018.05.171

[66] Duarte Alonso A Sakellarios N Cseh L. The theory of planned behavior in the context of a food and drink event: a case study. J Conv Event Tour. (2015) 16:200–27. doi: 10.1080/15470148.2015.1035822

[67] Horng J-S Su C-S So S-IA. Segmenting food festival visitors: applying the theory of planned behavior and lifestyle. Paper presented at the. J Conv Event Tour. (2013) 14:193–216. doi: 10.1080/15470148.2013.814038

[68] Shin YH Jung SE Im J Severt K. Applying an extended theory of planned behavior to examine state-branded food product purchase behavior: the moderating effect of gender. J Foodserv Bus Res. (2020) 23:358–75. doi: 10.1080/15378020.2020.1770043

[69] Su DN Johnson LW O’Mahony B. Will foodies travel for food? Incorporating food travel motivation and destination foodscape into the theory of planned behavior. Asia Pac J Tour Res. (2020) 25:1012–28. doi: 10.1080/10941665.2020.1805475

[70] Vesci M Botti A. Festival quality, theory of planned behavior and revisiting intention: evidence from local and small Italian culinary festivals. J Hosp Tour Manag. (2019) 38:5–15. doi: 10.1016/j.jhtm.2018.10.003

[71] Mumpower JL Liu X Vedlitz A. Predictors of the perceived risk of climate change and preferred resource levels for climate change management programs. J Risk Res. (2015) 19:798–809. doi: 10.1080/13669877.2015.1043567

[72] Aitken C Chapman R McClure J. Climate change, powerlessness and the commons dilemma: assessing new Zealanders’ preparedness to act. Glob Environ Chang. (2011) 21:752–60. doi: 10.1016/j.gloenvcha.2011.01.002

[73] Adam I. Backpackers’ risk perceptions and risk reduction strategies in Ghana. Tour Manag. (2015) 49:99–108. doi: 10.1016/j.tourman.2015.02.016

[74] Liu B Schroeder A Pennington-Gray L Farajat SA. Source market perceptions: how risky is Jordan to travel to? J Destin Mark Manag. (2016) 5:294–304. doi: 10.1016/j.jdmm.2016.08.005

[75] Chew EYT Jahari SA. Destination image as a mediator between perceived risks and revisit intention: a case of post-disaster Japan. Tour Manag. (2014) 40:382–93. doi: 10.1016/j.tourman.2013.07.008

[76] Carballo RR León CJ Carballo MM. The perception of risk by international travellers. Worldwide Hospit Tour Themes. (2017) 9:534–42. doi: 10.1108/WHATT-07-2017-0032

[77] Fuchs G Reichel A. An exploratory inquiry into destination risk perceptions and risk reduction strategies of first time vs. repeat visitors to a highly volatile destination. Tour Manag. (2011) 32:266–76. doi: 10.1016/j.tourman.2010.01.012

[78] Kim M Choi KH Leopkey B. The influence of tourist risk perceptions on travel intention to mega sporting event destinations with different levels of risk. Tour Econ. (2021) 27:419–35. doi: 10.1177/1354816619879031

[79] Olya HG Al-ansi A. Risk assessment of halal products and services: implication for tourism industry. Tour Manag. (2018) 65:279–91. doi: 10.1016/j.tourman.2017.10.015

[80] Wolff K Larsen S Øgaard T. How to define and measure risk perceptions. Ann Tour Res. (2019) 79:102759. doi: 10.1016/j.annals.2019.102759

[81] Chinazzi M Davis JT Ajelli M Gioannini C Litvinova M Merler S. The effect of travel restrictions on the spread of the 2019 novel coronavirus (COVID-19) outbreak. Science. (2020) 368:395–400. doi: 10.1126/science.aba9757 32144116 PMC7164386

[82] Lee C-K Song H-J Bendle LJ Kim M-J Han H. The impact of non-pharmaceutical interventions for 2009 H1N1 influenza on travel intentions: a model of goal-directed behavior. Tour Manag. (2012) 33:89–99. doi: 10.1016/j.tourman.2011.02.006, PMID: 32287736 PMC7115461

[83] Sánchez-Cañizares SM Cabeza-Ramírez LJ Muñoz-Fernández G Fuentes-García FJ. Impact of the perceived risk from COVID-19 on intention to travel. Curr Issue Tour. (2021) 24:970–84. doi: 10.1080/13683500.2020.1829571

[84] Choi J Lee A Ok C. The effects of consumers’ perceived risk and benefit on attitude and behavioral intention: a study of street food. J Travel Tour Mark. (2013) 30:222–37. doi: 10.1080/10548408.2013.774916

[85] Sparks B. Planning a wine tourism vacation? Factors that help to predict tourist behavioural intentions. Tour Manag. (2007) 28:1180–92. doi: 10.1016/j.tourman.2006.11.003

[86] Bond J Kriesemer S Emborg J Chadha M. Understanding farmers’ pesticide use in Jharkhand India. Exten Farm Syst J. (2009) 5:53.

[87] Bianchi C. Exploring urban consumers’ attitudes and intentions to purchase local food in Chile. J Food Prod Mark. (2017) 23:553–69. doi: 10.1080/10454446.2015.1048021

[88] Çabuk S Tanrikulu C Gelibolu L. Understanding organic food consumption: attitude as a mediator. Int J Consum Stud. (2014) 38:337–45. doi: 10.1111/ijcs.12094

[89] Karim MSA Chua B-L Salleh H. Malaysia as a culinary tourism destination: international tourists’ perspective. J Tour Hospit Culin Arts. (2009) 1:1–16.

[90] Akkuş G Erdem O. Food tourists’ intentions within the TPB framework (M00, M31). J Tour Gastron Stud. (2013) 3:9.

[91] Samdin Z Abdullah SINW Khaw A Subramaniam T. Travel risk in the ecotourism industry amid COVID-19 pandemic: ecotourists’ perceptions. J Ecotour. (2021) 21:266–294. doi: 10.1080/14724049.2021

[92] Memon MA Mirza MZ Lim B Umrani WA Hassan MA Cham TH. When in Rome, do as the romans do: factors influencing international students’ intention to consume local food in Malaysia. Br Food J. (2019) 122:1953–67. doi: 10.1108/BFJ-09-2018-0636

[93] Prapasawasdi U Wuttisittikulkij L Borompichaichartkul C Changkaew L Saadi M. Cultural tourism behaviors: enhancing the influence of tourists’ perceptions on local Thai Food and Culture. Open Psychol J. (2018) 11:184–97. doi: 10.2174/1874350101811010184

[94] Hamid S Azhar M. Behavioral intention to order food and beverage items using e-commerce during COVID-19: an integration of theory of planned behavior (TPB) with trust. Br Food J. (2022) 125:112–131. doi: 10.1108/BFJ-03-2021-0338

[95] Dedeoğlu SB Eren D Percin NS Aydin Ş. Do tourists’ responsible behaviors shape their local food consumption intentions? An examination via the theory of planned behavior. Int J Contemp Hospit Manag. (2022) 34:4539–4561. doi: 10.1108/IJCHM-05-2021-0579

[96] Fornell C. A national customer satisfaction barometer: the Swedish experience. J Mark. (1992) 56:6–21. doi: 10.1177/002224299205600103

[97] Khoshmaram M Shiri N Shinnar RS Savari M. Environmental support and entrepreneurial behavior among Iranian farmers: the mediating roles of social and human capital. J Small Bus Manag. (2020) 58:1064–88. doi: 10.1111/jsbm.12501

[98] Hair JF Hult GTM Ringle CM Sarstedt M Thiele KO. Mirror, mirror on the wall: a comparative evaluation of composite-based structural equation modeling methods. J Acad Mark Sci. (2017) 45:616–32. doi: 10.1007/s11747-017-0517-x

[99] Dijkstra TK Henseler J. Consistent and asymptotically normal PLS estimators for linear structural equations. Computat. Stat Data Anal. (2015) 81:10–23. doi: 10.1016/j.csda.2014.07.008

